# A Simple Restriction Fragment Length Polymorphism-Based Method for Multiplex Testing of Thrombosis Risk Factors FV Leiden and *F2* G20210A with Highly Sensitive Contamination Detection

**DOI:** 10.3390/ijms27010301

**Published:** 2025-12-27

**Authors:** Philippe de Mazancourt, Sylvie Grey, Elise Alabre, Mariam Keita, Jean-Pierre Rabès

**Affiliations:** 1 Laboratory of Biochemistry and Molecular Genetics, Ambroise Paré Hospital, GHU APHP-Université Paris-Saclay, F-92100 Boulogne-Billancourt, France; 2 UMR1179, INSERM, Université Versailles St-Quentin, F-78180 Montigny le Bretonneux, France; 3 Hematology Department, European Hospital Georges-Pompidou, GHU APHP-Centre-Université Paris Cité, F-75015 Paris, France

**Keywords:** molecular diagnosis, venous thromboembolism, genetic risk factors, FV Leiden, *F2* G20210A, restriction fragment length polymorphism, contamination detection

## Abstract

Factor V (FV) Leiden and *F2* G20210A are inherited genetic risk factors that are in the first line of laboratory tests for thromboembolic diseases. Their detection relies on PCR assays, which are subject to contamination, as well as pipetting error, when manually performed and require individual assays for each gene. In this article, we report an improved PCR and restriction endonuclease assay for the simultaneous detection of the FV Leiden and *F2* G20210A variants, based on multiplex amplification with fluorescent primers, digestion control, identity monitoring, and contamination tracking.

## 1. Introduction

Among the recognized inherited risk factors for venous thromboembolism (antithrombin, protein C and protein S deficiencies, dysfibrinogenemia, non-O blood group, elevated FVIII, Factor V Leiden (*F5*:c.1601G > A, p.Arg534Gln), and the *F2* G20210A variant (*F2*:c.*97G > A), only the latter two require DNA testing, whereas the former are assessed using functional and/or quantitative assays. Other inherited genetic risk factors have not been conclusively linked to an increased risk or require further validation, and are therefore not recommended as first-line laboratory tests [1,2,3,4]. However, Factor V (FV) Leiden and *F2* G20210A genetic testing are recommended in many clinical situations [5].

The initial methods for FV Leiden and *F2* G20210A testing relied on restriction endonuclease digestion assays (RFLP) [6,7]. The FV Leiden variant suppresses an MnlI cleavage site whereas the *F2* G20210A variant creates a HindIII site, provided that mutagenic primers are used. As there is no second HindIII site in the vicinity, digestion control can be added to the polymerase chain reaction (PCR) [8]. Multiplex assays for the simultaneous detection of both variants have been described [9,10].

Many other methods have been described, some of which are now rarely used, such as High-Resolution Melting (HRM) analysis or Single-Strand Conformation Polymorphism (SSCP) [11]. Allele-Specific PCR (AS-PCR) uses sequence-specific primers that selectively amplify either the wild-type or the mutant allele [12]. Locked Nucleic Acid (LNA) hybridization assay relies on the hybridization of multiplex-amplified fragments with biotin-labeled primers onto sequence specific oligonucleotides probes immobilized on nitrocellulose [13]. Although long considered the gold standard, Sanger sequencing is limited to specific indications because it is expensive and time-consuming. Detection through thrombophilia panels by Next Generation Sequencing (NGS) is highly efficient, but the cost is not covered by many health insurance programs, as the clinical relevance of many detected variants remains uncertain [1,2,5,14]. Currently, the most widely used assays rely on 5′ nuclease real-time PCR with fluorescent probes (e.g., TaqMan^®^), due to their potential for automation, high throughput, speed, and multiplexing [15]. However, this method is highly sensitive to the presence of variants in the probe hybridization sequence [16].

As the RFLP method is inexpensive, easy to set up, and suitable for low-throughput laboratories, it remains widely used. This study aimed to develop and validate a multiplex fluorescent PCR–RFLP for the simultaneous detection of the FV Leiden and *F2* G20210A variants. We added internal controls for digestion efficiency, identity monitoring and contamination tracking that fills a gap in commercial kits. The method is based on multiplex amplification with fluorescent primers targeting *F5* exon 10, the *F2* 3′UTR, the fibrinogen alpha gene (*FGA*) intron 1 for HindIII digestion control, and two short tandem repeats (STRs; SE33 and FGA) for identity monitoring and contamination tracking.

## 2. Results

### 2.1. Primer Design, Initial Set up and Validation

The primers and fluorochrome labels are described in the Materials and Methods section. The FV Leiden variant suppresses an MnlI restriction site. The *F5* exon 10 fragment also carries a mandatory additional MnlI site used as an internal digestion control.

The HindIII site specific to the *F2* G20210A variant was created by a mutagenic primer as previously described [6,7]. The upper primer was repositioned to avoid the amplification of an MnlI site. HindIII-undigested fragments might result from accidentally undigested fragments rather than from the absence of restriction sites. A digestion control was therefore added, as the putative HindIII restriction site is unique within the *F2* amplified fragment. For HindIII control fragment amplification, the primers described in [8] could not be used because the PCR product contains an MnlI site. Instead, an *FGA* intron 1 fragment carrying a mandatory HindIII site and no MnlI site was added to the multiplex PCR as a HindIII digestion control.

For identity monitoring, microsatellites were preferred over a panel of SNPs. Amplifying just a few microsatellite loci is sufficient because of their high level of polymorphism. Their ease of analysis by PCR and their straightforward detection during electrophoresis, combined with their high polymorphism rate, help prevent sample mix-ups and allow the detection of contaminations. (see also Section 2.5). The FGA and SE33 STRs (GenBank accession M64982 and V00481) are complex tetranucleotide repeats initially described in DNA fingerprinting applications. Primers were adapted from [16]. With the chosen primers, MnlI cuts the STR of *FGA*. To overcome this difficulty, the fluorescent labeling was located on the upper primer, still allowing fluorescent STR analysis after MnlI digestion. Since the size of the amplified fragments varies widely, it was important to use a distinct fluorochrome to ensure clear differentiation of the STRs from the HindIII control fragment and from the *F5* and *F2* gene segments. The amplicon sizes are described in Table 1.

**Figure 1:**
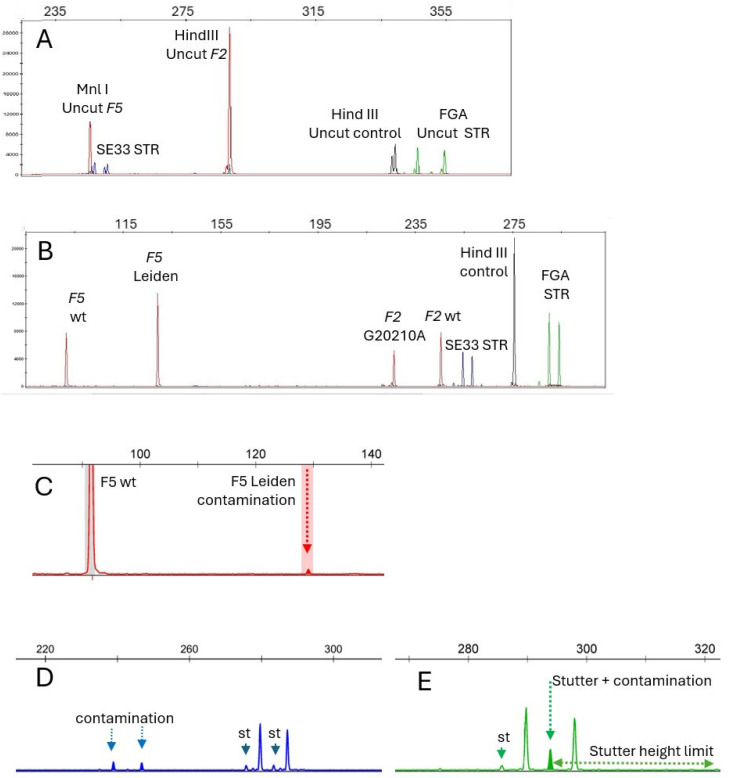
Undigested sample, double heterozygous sample, and contaminated sample. 1 µL of polymerase chain reaction (PCR) product was added to 9 μL Hi-Di formamide and 0.5 μL size standard (GS™-LIZ^®^ 600, Applied Biosystems). The mix was then loaded onto a capillary electrophoresis apparatus (Applied Biosystems 3500 xL Dx). The horizontal scale indicates apparent fragment sizes in base pairs. (Panel (**A**)). Undigested PCR sample: the undigested HindIII NED-labeled control fragment is present (Hind III Uncut control, in black). The MnlI undigested FV fragment is present (MnlI Uncut F5, in red). (Panel (**B**)). Electropherogram of a double heterozygous sample (FV Leiden +/− and F2 G20210A +/−). (**A**): PET-labeled F2 and F5 fragments (red) digested with Mnl I and Hind III, respectively. The NED-labeled FGA intron I (green) serves as the Hind III digestion control (expected size 275 bp). The uncut FGA intron 1 fragment (339 pb) and the uncut FV fragment (288 pb) are both absent. The different size for VIC-labeled PCR products (FGA STR alleles, green) and 6-FAM-labeled PCR products (SE33 STR alleles, blue) in panels (**A**–**C**) reflects the different sample genotypes. (Panel (**C**)): Experimental dilution 1:20 of a heterozygous FV Leiden individual’s DNA into a wild-type DNA. The DNA sample used for experimental contamination was deliberately chosen to carry the FV-Leiden variant. The FV Leiden+/− and F2 G20210A −/− was mixed (1:20 ratio) in a FV-Leiden−/− and F2 G20210A −/− DNA sample. The dotted arrow shows the peak resulting from contamination. (Panel (**D**)): Zoom in on contamination and stutter peaks at the SE33 locus. Stutters (st) are indicated by plain arrows. Contamination is detected by the presence of extra SE33 peaks (dotted line arrows). Additional STR alleles (dotted arrows) correspond to those of the contaminating DNA. (Panel (**E**)): Zoom in on contamination and stutter peaks at the FGA locus. Stutters (st) are indicated by plain arrows. Contamination by an allele that matches the stutter size (in bp) overlaps the stutter peak. Note that contamination would still be detected because it exceeds the expected stutter height (11% of the main peak).

The co-amplification and co-digestion conditions are described in the Materials and Methods section. After testing the initial mix, we concluded that it was necessary to adjust individual primer pair concentrations to limit unbalanced amplification (see Table 2 for final primer concentrations). These primer sequences and concentrations, along with the optimized amplification and digestion conditions, ensured reliable multiplex amplification, effective digestion control and robust contamination tracking (see also Figure 1). 

### 2.2. Accuracy

No discrepancies were observed in a series of samples analyzed by either dideoxy sequencing or next-generation sequencing, compared to the improved RFLP method described in this article (heterozygous *F2* G20210A: n = 14 cases; wild-type: n = 31 cases; homozygous FV Leiden: n = 1 case; and heterozygous FV Leiden: n = 16 cases), see Table 2. Since no amplification failures occurred and all results were concordant, both specificity and sensitivity were 100%. The genotypes were tested in different runs (bidirectional sequencing, single determination; 48 patients analyzed across four different runs for Sanger analysis) or were selected from carriers identified by NGS analysis (single determination, paired-strand analysis; 14 patients selected across seven different runs). Total accuracy, positive predictive value and negative predictive value were 100%, see Table 3.

### 2.3. Sensitivity

DNA concentration was photometrically determined. DNA from two individuals were diluted stepwise in sterile water, to achieve concentrations corresponding to 2, 4, 8, 16, 32, 64, 128, and 256 ng in a 10 µL PCR. Under these conditions, with as little as 2 ng of input DNA, all peaks were present. When DNA quantities were in the 15–70 ng range per 10 µL reaction—which is typically achieved with the laboratory’s standard methods—satisfactory signal height and peak balance were obtained with 28 cycle amplifications (see Materials and Methods for amplification conditions). Thus, after the initial set up experiments, DNA concentration was no longer measured, and DNA input was set to 2 µL regardless of the actual concentration. A representative electropherogram is shown in Figure 1.

### 2.4. Additional Validation Steps

The validation steps were performed as required by the ISO 15189 certification [17]. These validation steps were performed as recommended (https://anpgm.fr/recommandations-professionnelles/, accessed on 28 September 2025).

Formal limit of detection: An inherent fluorescent background is present in all capillary electrophoresis runs. Fluorescence noise was defined as the highest background peak observed in blank assays loaded in formamide. Under our experimental conditions, the noise was below 50 relative fluorescent units (RFU). A cut-off value of 150 RFU (three times the noise) was therefore introduced to facilitate the interpretation and reliable identification of true alleles in the optimized assay.

Incomplete digestion tracking was performed by checking the absence of 289 and 339 bp fragments for undigested MnlI and HindIII PCR fragments (Figure 1A,B). No incomplete digestion or assay drift was observed across 10 independent runs.

Inter-assay reproducibility was verified by including the same control DNAs in each run (one individual without variants, one individual carrying both heterozygous variants, and one individual homozygous for the FV Leiden variant). Since these controls are included in every series, they are also used to monitor both inter- and intra-operator reproducibility (100% concordance for three operators, with 10 independent runs performed by each operator).

Inter-operator variability was assessed by repeating the analysis of two randomly selected DNA samples that had been analyzed by a different operator in a previous run. In total, 60 samples across 30 independent runs were evaluated (three operators, 10 independent runs each). The assay demonstrated 100% overall agreement.

*F5* and *F2* allele peak height ratios: for 30 heterozygous control patients evaluated in 30 different runs over a 2-year period, the peak height ratios for variant to wild-type alleles were 0.61 ± 0.06 for the *F5* alleles and 0.67 ± 0.09 for the *F2* alleles, respectively (mean ± standard deviation [SD], n = 30). Complete digestion was confirmed by complete digestion of the HindIII control fragment. Thus, for heterozygous carriers, any peak height ratio falling below mean values ± 2 SD (0.49 for *F5* and 0.49 for *F2*, respectively) would be reanalyzed.

### 2.5. Contamination Detection and Stutter Peaks

The dual-STR control system ensures both sample traceability and contamination detection, offering a clear improvement over traditional single-marker method. FGA and SE33 STRs were chosen because of their high heterozygosity percentage and high allelic heterogeneity rate. For FGA, there are over 28 known alleles ranging from 12.2 to 51.2 repeats and over 76 known alleles ranging from 7 to 39.2 repeats for SE33. The random match probability for the combination of the most frequent genotypes is in the 0.0005 range with these two STRs. The inclusion of such STRs in the multiplex mix enabled quality control and the identification of verifications. Moreover, the duplicate distribution of a given sample instead of two different samples would be detected. STR co-amplification allowed for contamination detection by highlighting any additional STR peak (Figure 1C,D), regardless of the mechanism involved, whereas traditional methods would only detect reagent contaminations through signal presence in the blank control reaction.

This method amplifies STR-generated artifact stutter peaks in strict repetitive patterns to a size equivalent to alleles one repeat shorter than the respective allelic types. They result from polymerase slippage during template amplification [18]. In a series of 30 individuals randomly selected in six different assays on the same instrument, stutter heights were 7.6 ± 1.8% and 7.6 ± 1.6% of the allele height for FGA and SE33, respectively (mean ± SD). Thus, in ongoing assays, any stutter peak above the mean +2 SD (i.e., 11%) values will be considered as resulting from contamination.

To assess the potential for contamination detection, thirty samples (FV Leiden −/− and F2 G20210A −/−, n = 14; FV Leiden +/− and F2 G20210A −/−, n = 5; FV Leiden −/− and F2 G20210A +/−, n = 3) with DNA concentrations ranging from 24 to 79 ng/µL were combined to generate 30 mixed DNA samples at 1:10 and 1:20 (ng/ng) ratios (Table 4). Mixed DNAs at 1:20 ratio allowed for contamination identification either by additional STR or variant alleles (Figure 1C–E), by a stutter size above the expected value, or by an abnormal allelic F5 or F2 peak height ratio. Further dilutions usually generated peaks below the interpretation limit (150 RFU).

## 3. Discussion

This method is affordable, cheaper than TaqMan-based methods, easy to set up in any laboratory, specific, and sensitive. It has the potential for scalability or integration into automated workflows. In essence, the modifications involved the detection in a single PCR of *F2* G20210A and FV Leiden genotypes in multiplex amplification with modified primer sets to overcome cross digestions. Although methods must always be adjusted to each laboratory, the process reported in this article may be useful for other laboratories wishing to adapt this assay. This method reduces the mistyping risk, either by contamination or interference with other SNPs. Addition of internal control for digestion and STR co-amplification enables contamination detection and possible sample mix-ups. These advantages overcome the disadvantage of the method being a three-step assay (amplification, digestion, and electrophoresis) compared to single-step TaqMan-based assays.

There are some limitations: We cannot provide quantitative data for the contamination detection threshold, since many mechanisms may contribute to peak imbalance, with the most common being the preferential amplification of small alleles or the preferential injection of small alleles in overloaded PCR samples [19]. Moreover, under borderline contamination conditions, the detection of contaminating DNA depends both on the total amount of DNA in the PCR and on the DNA ratio. Not detecting significant contamination would require the contaminating genotype to have all four alleles matching either the allele sizes or would require the stutter positions of the sample being contaminated. Although contamination with a misleading genotype is very unlikely, we added a last control; if the peak height ratio falls below 50% for a heterozygous FV Leiden or F2 G20210A genotype, the analysis would be repeated even in the absence of an additional STR peak.

In conclusion, FV Leiden and *F2* G20210A are inherited genetic risk factors that are in the first line of laboratory tests for thromboembolic diseases. The detection of FV Leiden and *F2* G20210A relies on PCR assays that are subject to contamination and pipetting errors. The present report describes a multiplex assay for co-amplification of FV Leiden and *F2* G20210A. Additional co-amplified controls detect digestion failure. Microsatellites in the PCR enable the detection of contamination and identity errors for each analyzed sample. Previously reported PCR-RFLP methods do not allow for the sample-by-sample tracking of contamination and identity errors. NGS is the only method that allows for the tracking of contamination and identity errors; however, it is time-consuming and costly. Therefore, NGS is not performed as a first-line genetic test for thrombotic events. The PCR-RFLP method described here offers a robust, low-cost alternative for routine thrombophilia screening with integrated contamination safeguards.

## 4. Materials and Methods

### 4.1. DNA Samples

DNA samples were obtained from patients that were referred for FV Leiden and *F2* G20210A detection by the standard procedure in the lab [6,8]. All patients signed an informed consent allowing for the use of their DNA for method set up and research.

DNA was extracted using fast extraction protocols, such as the QuickGene Blood DNA extraction kit from Qiagen, Hilden, Germany (expected value in the 25 ng/µL range), or the Maxwell RSC whole-blood DNA kit from Promega, Madison, WI, USA (expected values in the 20–80 ng/µL range).

### 4.2. Primer Design

Design of the PCR restriction fragment length polymorphism (RFLP)-based method: Primers were designed with the primer3 software (https://primer3.ut.ee/, accessed on 17 November 2025) and carefully chosen among the proposed sets, based on the absence (or very low frequency) of single-nucleotide polymorphisms (SNPs) in the hybridization sequences at the 3′ end of the primers. Primers were chosen to hybridize with SNP-free sequences or in the 0.0005 frequency range (https://genome.ucsc.edu/cgi-bin/hgGateway, accessed on 17 November 2025); see Table 5 for primer sequences and labeling.

### 4.3. PCR Conditions

PCR conditions and digestion were as follows: amplifications (MasterMix PCR AmpliTaq Gold360 from Thermofisher Scientific, Waltham, MA, USA, ref 4398876) were performed in a 10 µL final volume with 15–50 ng DNA per tube. Primer sequences and concentrations were as indicated in Table 5. Initial denaturation at 95 °C for 10 min was followed by 28 annealing cycles at 60 °C for 30 s, extension at 72 °C for 60 s, and denaturation at 95 °C for 30 s, with a final round at 72 °C for 7 min. Each primer pair was tested in individual reactions on control DNAs before being mixed in multiplex reactions. After testing the initial mix, we concluded that it was necessary to adjust individual primer pair concentrations to limit unbalanced amplification (see Table 5 for primer concentrations).

### 4.4. Restriction Digestion

Co-digestion was carried out overnight with MnlI and HindIII (1 and 3 UI/µL final, respectively). Co-digestion was optimized by choosing a manufacturer carrying both enzymes and a universal digestion buffer (New England Biolabs, Evry, France, ref R063 for MnlI and R0104 for HindIII, respectively). The enzymes and buffer were premixed prior to distribution to lower the pipetting error risk.

### 4.5. Capillary Electrophoresis and Data Analysis

After enzymatic digestion, 1 µL of polymerase chain reaction (PCR) product was added to 9 μL Hi-Di formamide (Applied Biosystems) and 0.5 μL size standard (GS™-LIZ^®^ 600, Applied Biosystems), and then loaded onto a capillary electrophoresis apparatus (Applied Biosystems 3500 xL Dx). Allele calling was performed with GeneMapper^®^ Software version 5.0 (Thermofisher Scientific, ref A38892). With as low as 2 ng of input DNA, all peaks were in the 250–32,000 relative fluorescent units (RFU) range, 32,000 RFU being the saturation limit and 250 RFU the arbitrary detection threshold (cutoff for allele calling).

### 4.6. Genotype Verifications

All genotypes were confirmed by NGS or dideoxy-sequencing. Primers for amplification and Sanger sequencing were 5′acatggttaaggcctgttgc and 5′gccacatctggcttgaaatt for *F5* exon 10, and 5′tggcaagggtaaacagatcc and 5′ctcaggcactcctctcaacc for *F2* 3′UTR. Amplifications were performed with AmpliTaq Gold 360 mastermix (Thermofisher Scientific, ref 4398876). Sequencing was performed with the BigDye Terminator reagents v.1.1 cycle sequencing reagents (Applied biosystems Courtaboeuf, France, ref 4486480) according to manufacturer protocols and loaded onto a capillary electrophoresis apparatus (Applied Biosystems 3500xL-Dx). NGS was performed with a capture custom panel including exon sequences of 55 genes–including *F5* and *F2*—designed by Twist Bioscience (San Francisco, CA, USA). Captured libraries (Twist Library Preparation Enzymatic Fragmentation Kit, Twist Bioscience, ref 104175) were loaded onto a MiSeq sequence analyzer (Illumina, Evry, France).

## Figures and Tables

**Table 1:** Expected size of the fragments.

*F5* wt	*F5* Leiden	F5 MnlI Uncut	*F2* wt	*F2* G20210A	HindIII Control Uncut	HindIII Control	FGA	SE33
92	129	*289* *	246	226	*339* **	276	92–243 ***	279–421

Apparent sizes (in base pairs) with the mobility G5 set and fluorochromes PET, FAM, VIC and NED on an Applied 3500xL-Dx sequence analyzer with POP7 in 50 cm capillaries. *: must completely disappear after MnlI digestion. **: must completely disappear after HindIII digestion. ***: expected fragment sizes without MnlI digestion: 148–297 bp. The expected fragment sizes for FGA and SE33 STRs correspond to the allele ranges of 14 to 50.2 repeats for FGA and 4.2 to 39 repeats for SE33, respectively.

**Table 2:** Concordance study of the multiplex RFLP genotyping test and sequence analysis.

FV Leiden	*F2* G20210	n	Concordance
−/−	−/−	31	34/34 (100%)
+/−	−/−	16	22/22 (100%)
−/−	+/−	14	4/4 (100%)
+/+	−/−	1	1/1 (100%)
All genotypes		62	62/62 (100%)

Genotype of 62 individuals genotyped by the RFLP method described in this paper, and either Sanger or NGS. −/−: absence of the variant, +/−: heterozygous individuals.

**Table 3:** Sensitivity and specificity of the multiplex RFLP genotyping test.

FVL-F2-Genotyping Test	Sensitivity (95% CI) ^a^	Specificity (95% CI) ^a^
Multiplex RFLP	1.0 (0.94–1.0)	1.0 (0.93–1.0)
Sanger or NGS	1.0 (0.94–1.0)	1.0 (0.93–1.0)

^a^: The sensitivity and specificity were calculated by assuming that the sequencing analysis of the 62 clinical samples was correct. True positive individuals are the ones with concordance on both F2 and FV Leiden genotypes.

**Table 4:** Detection of added diluted DNA in samples.

Samples	*F5* −/−; *F2* −/−	*F5* +/−; *F2* −/−	*F5* −/−; *F2* +/−
Added DNA ratio 1:10 (ng)	*F5* −/−; *F2* −/− n = 11 Detected: 11 (100%)	*F5* −/−; *F2* −/− n = 5 Detected: 5 (100%)	*F5* −/−; *F2* −/− n = 5 Detected: 5 (100%)
	*F5* +/−; *F2* −/− n = 7 Detected: 7 (100%)	*F5* +/−; *F2* −/− n = 0 -	*F5* +/−; *F2* −/− n = 0 -
	*F5* −/−; *F2* +/− n = 2 Detected: 2 (100%)	*F5* −/−; *F2* +/− n = 0 -	*F5* −/−; *F2* +/− n = 0 -
Total samples 30; added DNA (1:10) detected 30 (100%)			
Sensitivity (95% CI): 1.0 [0.89–1.0]		Specificity (95% CI) 1.0 [0.89–1.0]	
Samples	*F5* −/−; *F2* −/−	*F5* +/−; *F2* −/−	*F5* −/−; *F2* +/−
Added DNA Ratio 1:20 (ng)	*F5* −/−; *F2* −/− n = 11 Detected: 11 (100%)	*F5* −/−; *F2* −/− n = 5 Detected: 5 (100%)	*F5* −/−; *F2* −/− n = 5 Detected: 5 (100%)
	*F5* +/−; *F2* −/− n = 7 Detected: 7 (100%)	*F5* +/−; *F2* −/− n = 0 -	*F5* +/−; *F2* −/− n = 0 -
	*F5* −/−; *F2* +/− n = 2 Detected: 2 (100%)	*F5* −/−; *F2* +/− n = 0 -	*F5* −/−; *F2* +/− n = 0 -
Total samples 30; added DNA (1:20) detected 30 (100%)			
Sensitivity (95% CI): 1.0 [0.89–1.0]		Specificity (95% CI) 1.0 [0.89–1.0]	

−/−: absence of the variant, +/−: heterozygous individuals. Thirty samples (FV Leiden −/− and *F2* G20210A −/−, *n* = 14; FV Leiden +/− and *F2* G20210A −/−, *n* = 5; FV Leiden −/− and F2 G20210A +/−, *n* = 3) with DNA concentrations ranging from 24 to 79 ng/µL were combined to generate 30 mixed DNA samples at 1:10 and 1:20 (ng/ng) ratios. Contamination can be detected by the presence of an additional STR allele (allele-calling threshold: 150 RFU), by an STR stutter peak exceeding the mean + 3 SD of the corresponding allele (expressed as a percentage), or by an imbalanced wild-type/variant allele peak height ratio < 0.49. If any of these three criteria is met, the sample should be considered to contain additional DNA.

**Table 5:** Primers and polymerase chain reaction (PCR) conditions.

PCR Primers	5′ Label	Sequence (5′ ≥ 3′)	Final (µM)
*F5* Exon 10 Upper	-	GGAACAACACCATGATCAGAGCA	1
*F5* Exon 10 Lower	PET	TAGCCAGGAGACCTAACATGTTC	1
*F2* 3′UTR Upper	PET	GGTATCAAATGGGCATCGTC	1
*F2* 3′UTR Lower	-	ATAGCACTGGGAGCATTGA**A**GC	1
STR SE33 Upper	6-FAM	AATCTGGGCGACAAGAGTGA	2
STR SE33 Lower	**-**	ACATCTCCCCTACCGCTATA	2
STR FGA Upper	VIC	GGCTGCAGGGCATAACATTA	1
STR FGA Lower	**-**	ATTCTATGACTTTGCGCTTCAGGA	1
*FGA* intron 1 Upper	NED	ACTGAAGCAGCAATTACAGGAG	2
*FGA* intron 1 Lower	-	AAGTCCCCAGGAAGAGATGG	2

The mutagenic base in the lower *F2* primer is presented with underlined bold characters. As primers were chosen to generate fragments with markedly different sizes, many fluorochrome combinations are possible. The one reported in this article uses a combination of PET, VIC FAM, and NED, but ATTO 565, Yakima Yellow, FAM and ATTO 550 (red, green, blue, and black, respectively, with the Applied G5 filter) would be an equivalent combination. Since equimolar primer concentrations resulted in unbalanced fragment amplification, the primer concentrations were adjusted accordingly. The values provided in Table 5 are those used in routine testing.

## Data Availability

The original contributions presented in this study are included in the article. Further inquiries can be directed to the corresponding author.
